# When timing and dose of nutrition support were examined, the modified Nutrition Risk in Critically Ill (mNUTRIC) score did not differentiate high-risk patients who would derive the most benefit from nutrition support: a prospective cohort study

**DOI:** 10.1186/s13613-018-0443-1

**Published:** 2018-10-22

**Authors:** Charles Chin Han Lew, Gabriel Jun Yung Wong, Ka Po Cheung, Robert J. L. Fraser, Ai Ping Chua, Mary Foong Fong Chong, Michelle Miller

**Affiliations:** 1 Nutrition and Dietetics, College of Nursing and Health Sciences, Flinders University, GPO Box 2100, Adelaide, SA 5001 Australia; 2 Dietetics and Nutrition Department, Ng Teng Fong General Hospital, 1 Jurong East Street 21, Singapore, 609606 Singapore; 3 Department of Gastroenterology and Hepatology, College of Medicine and Public Health, Flinders University, GPO Box 2100, Adelaide, SA 5001 Australia; 4 Department of Respiratory Medicine, Ng Teng Fong General Hospital, 1 Jurong East Street 21, Singapore, 609606 Singapore; 5 Saw Swee Hock School of Public Health, National University of Singapore, 12 Science Drive 2 #10-01, Singapore, 117549 Singapore

**Keywords:** NUTRIC, Mortality, Nutrition support, Critical illness

## Abstract

**Background:**

The timing and dose of exclusive nutrition support (ENS) have not been investigated in previous studies aimed at validating the modified Nutrition Risk in Critically Ill (mNUTRIC) score. We therefore evaluated the mNUTRIC score by determining the association between dose of nutrition support and 28-day mortality in high-risk patients who received short- and longer-term ENS (≤ 6 days vs. ≥ 7 days).

**Methods:**

A prospective cohort study included data from 252 adult patients with > 48 h of mechanical ventilation in a tertiary care institution in Singapore. The dose of nutrition support (amount received ÷ goal: expressed in percentage) was calculated for a maximum of 14 days. Associations between the dose of energy (and protein) intake and 28-day mortality were evaluated with multivariable Cox regressions. Since patients have different durations of ENS, only the first 6 days of ENS in patients with short- and longer-term ENS were assessed in the Cox regressions to ensure a valid comparison of the associations between energy (and protein) intake and 28-day mortality.

**Results:**

In high-risk patients with short-term ENS (*n* = 106), each 10% increase in goal energy intake was associated with an increased hazard of 28-day mortality [adj-HR 1.37 (95% CI 1.17, 1.61)], and this was also observed for protein intake [adj-HR 1.31 (95% CI 1.10, 1.56)]. In contrast, each 10% increase in goal protein intake in high-risk patients with longer-term ENS (*n* = 146) was associated with a lower hazard of 28-day mortality [adj-HR 0.78 (95% CI 0.66, 0.93)]. The mean mNUTRIC scores in these two groups of patients were similar.

**Conclusion:**

When timing and dose of nutrition support were examined, the mNUTRIC did not differentiate high-risk patients who would derive the most benefit from nutrition support.

**Electronic supplementary material:**

The online version of this article (10.1186/s13613-018-0443-1) contains supplementary material, which is available to authorized users.

## Background

Non-volitional nutrition support is frequently required in the intensive care unit (ICU). This seemingly straightforward therapy has garnered increased attention in the literature reflecting conflicting evidence surrounding the optimal timing, dose, rate of advancement, and composition of nutrition support [1]. While several studies demonstrated delayed [2, 3] or permissive underfeeding [4–6] to be either benign or beneficial, these modes of feedings have been reported to be detrimental in other studies [7–9]. A possible explanation for the disparate findings is that a one-size-fits-all approach to nutrition support is not applicable to the needs of a heterogeneous group of critically ill patients [1].

To address this issue, Heyland et al. [10] developed a score [Nutrition Risk in Critically Ill (NUTRIC)] to better determine patients in a heterogeneous ICU population who would be more likely to benefit from adequate nutrition support. While the original score comprised six components, it was subsequently revised to exclude interleukin-6 concentrations as this is rarely measured outside of research settings [9]. Consequently, the modified NUTRIC (mNUTRIC) has five components [i.e. age, Acute Physiology and Chronic Health Evaluation II (APACHE II), Sequential Organ Failure Assessment (SOFA), number of comorbidities, and days in hospital before admission to ICU] with scores “0–4” and “5–9” classified as low risk and high risk, respectively [11, 12]. There are now four published validation studies [9, 11–13] with three showing acceptable external validity for the mNUTRIC score: high-risk patients who received higher average energy [9, 11, 12] and protein [11] intake were observed to have lower mortality. These results suggested that goal energy and protein intake should be achieved as soon as possible via early aggressive (i.e. high dose) nutrition support including: (1) starting enteral feeding at goal rate [14], (2) using prokinetic agents prophylactically to enhance enteral feeding tolerance [15], and/or (3) using supplemental or total parenteral nutrition support when enteral nutrition cannot meet requirements within the first few days of ICU admission [7, 16].

However, recent evidence conflicts with these aggressive feeding practices. In patients with acute respiratory distress syndrome (ARDS), Braunschweig et al. [17] and Peterson et al. [18] reported that early aggressive nutrition support with a higher energy intake at the most acute phase of critical illness (i.e. around day 1 to day 7 of ICU admission) was associated with increased mortality. A close examination on the patients’ characteristics of Braunschweig et al. [17] revealed that most would be classified as high risk by the mNUTRIC (score = 5 since mean age, SOFA, APACHE II, and length of hospitalisation before ICU admission were 59 years old, 8.3, 22.5, and 3 days, respectively). In addition, Doig et al. [19] showed that early aggressive feeding at the initial stage of ICU admission was associated with higher mortality in patients with refeeding syndrome (even with adequate phosphate replacement). These studies suggest that early aggressive nutrition support may not benefit all critically ill patients.

To date, the mNUTRIC recommendations would support early aggressive nutrition treatment for high-risk patients, but the concerns of harm associated with early aggressive nutrition call into question the generalisability of the mNUTRIC score to all patients. It is therefore timely to re-evaluate if early aggressive nutrition support is of benefit to all high-risk patients in a heterogeneous ICU. Since the effects of timing and dose of nutrition support have not been investigated in previous mNUTRIC studies, we therefore aimed to determine whether timing and dose of nutrition support in critically ill patients may modify the association between mNUTRIC categories (low risk and high risk) and 28-day mortality in a single-centre cohort study.

## Methods

### Patient and setting

This was a prospective observational cohort study conducted in a 35-bed ICU in Ng Teng Fong General Hospital (Singapore) between August 2015 and October 2016. The ICU functions as a closed unit where board-certified intensivists and residents provide care for both medical and surgical patients. Treatment bias was minimised by blinding the intensivists and nurses to the objectives of the study.

To determine whether the association between mNUTRIC categories and 28-day mortality was modified by not only the dose of nutrition support (as in the original study), but also its timing, all patients ≥ 21 years old and had > 48 h of mechanical ventilation and enteral or parenteral feeding planned were included in the study. In addition, these patients were not declared moribund by an intensivist and had nutritional status determined by a dietitian (using the Subjective Global Assessment [20]) within 48 h of ICU admission. Nutritional status was an inclusion criterion because it has been previously associated with mortality in ICU patients [21–23].

As per usual clinical practice in our unit, all patients received a nutritional assessment and were prescribed an appropriate enteral or parenteral feeding regime within 48 h of ICU admission. As the mNUTRIC was not part of the routine nutritional assessment, it was calculated at the end of the study to minimise treatment bias. For the calculation of energy and protein goals, actual body weight taken at ICU admission using weighing bed was used. In obese patients (body mass index > 30 kg/m^2^), adjusted body weights [(actual body weight − ideal body weight) × 0.25 + ideal body weight] [24] were used. The dose of enteral and/or parenteral formulas received was recorded in the electronic medical records and verified by the attending nurse at the end of each shift.

### Data collection

All data [i.e. demographics, disease severity scores (e.g. APACHE II), comorbidities, baseline nutritional status, admission diagnoses, medications, intravenous fluids, energy and protein provided by enteral and/or parenteral nutrition, and clinical outcomes] were prospectively measured and recorded in the electronic medical records. The daily energy and protein intake of patients was calculated while receiving exclusive nutrition support (ENS), either enterally and/or parenterally, from ICU admission to a maximum of 14 days, unless death occurred earlier. Energy and protein intake was calculated from enteral formulas, protein modular and ready-to-use or compounded parenteral formulas. In addition, energy provided by propofol and dextrose-containing intravenous fluids were included in the calculation of total energy intake.

The dose of nutrition support was calculated by dividing the total energy and protein received by the number of days on ENS, and expressed as a percentage of the goals established at baseline [25]. Nutrition support received on the day of death was excluded in the calculation of total energy and protein intake since patients would not have received the entire prescription [10]. Ethics approval was granted by the Domain Specific Review Board (NHG DSRB Ref: 2014/00878).

### Statistical analysis

The association between energy (and protein) intake and 28-day mortality was examined in two sets of multivariable Cox proportional hazard regressions. In the first set, we examined the association between each 10% increase in goal energy (and protein) intake and 28-day mortality for the entire cohort. In the second set, the effects of timing and dose of nutrition support on 28-day mortality were examined. Braunschweig et al. [17] and Peterson et al. [18] observed that the dose of energy intake at the early phase of critical illness (ICU day 1 to day 7) was positively associated with mortality, and a crossover effect was observed at the later phase in patients who required longer-term nutrition support (ICU day 8 onwards). Therefore, we determined whether this phenomenon was also present in our cohort by calculating the average goal per cent energy and protein intake from day 2 (mean of day 1 and day 2) to day 14 (mean of day 1 to day 14) in survivors and non-survivors and plotting their relationships stratified by mNUTRIC categories (low and high risk). Thereafter, we defined “short-term ENS” and “longer-term ENS” intervals by observing for crossover associations between the per cent goal energy (and protein) intake in survivors and non-survivors.

The associations between energy (and protein) intake and 28-day mortality were determined by multivariable Cox proportional hazard regression. Covariates to be adjusted were identified by comparing the patients’ baseline characteristics using Student’s *t* test, Chi-square test, or Mann–Whitney *U* test as appropriate. Characteristics that were significantly different (*p* value < 0.05) between survivors and non-survivors at the univariate level were included as covariates in the multivariable Cox proportional hazard regressions to generate the adjusted hazard ratio (adj-HR). Variance inflation factors and tolerances were used to check for multicollinearity. The above steps were repeated in multivariable logistic regressions to generate figures that depict the associations between goal energy (and protein) intake and 28-day mortality (Additional file 1: Table S1 and Figure S1). Statistical analyses were performed using STATA 14.2 (StataCorp, College Station, TX, USA). For all comparisons, associations, and interactions, *p* value < 0.05 was considered as significant.

## Results

**Fig. 1:**
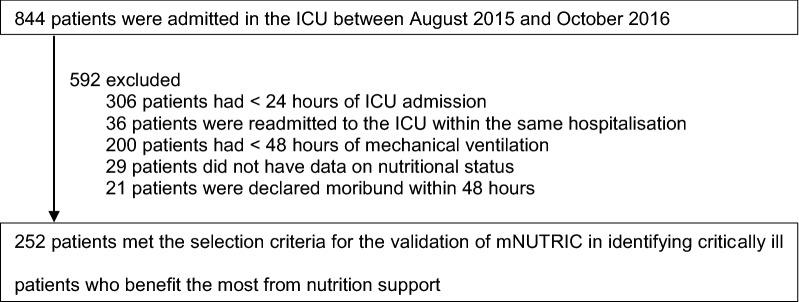
Enrollment of patients

There were 252 patients enrolled (Fig. 1), and no patients were lost to follow-up. Mortality at day 28 following ICU admission was 33.3%, and the characteristics of survivors and non-survivors are summarised in Table 1. Non-surviving patients had a significantly higher mNUTRIC score, were more likely to be malnourished, admitted for medical reasons, transferred from the general ward, and resuscitated before ICU admission. Patients who were excluded had similar characteristics to those enrolled apart from a lower SOFA score (median 8 vs. 9, *p* < 0.001) and a higher number of comorbidities (median 3 vs. 2, *p* < 0.001).

**Table 1:** Comparison of characteristics between 28-day survivors and non-survivors as well as patients who received short-term and longer-term exclusive nutrition support

Patients’ characteristics	Survivors (*n* = 168)	Non-survivors (*n* = 84)	*p*	Short-term ENS (≤ 6 days) (*n* = 106)	Longer-term ENS (≥ 7 days) (*n* = 146)	*p*
Age (years)	56.6 (15.6)	66.5 (15.1)	< 0.001	60.4 (16.9)	59.5 (15.5)	0.655
Male	108 [64.3]	47 [56.0]	0.200	63 [59.4]	92 [63.0]	0.564
BMI (kg/m^2^)	24.5 (21.6, 28.5)	24.7 (22.1, 29.0)	0.673	24.5 (21.2, 29.5)	24.6 (21.8, 28.4)	0.726
Location before adm			0.002			0.976
ED/HD/OT	148 [88.1]	61 [72.6]		88 [83.0]	121 [82.9]	
Wards	20 [11.9]	23 [27.4]		18 [17.0]	25 [17.1]	
Type of adm			0.001			0.001
Medical	94 [56.0]	65 [77.4]		80 [75.5]	79 [54.1]	
Surgery	74 [44.0]	19 [22.6]		26 [24.5]	67 [45.9]	
No. of comorbidities	2.0 (1.0, 3.0)	3.0 (2.0, 4.0)	0.007	3.0 (1.0, 4.0)	2.0 (1.0, 3.0)	0.002
LOS before ICU adm (days)	0.0 (0.0, 1.0)	1.0 (0.0. 2.0)	0.068	0.0 (0.0, 1.3)	0.0 (0.0, 1.3)	0.730
APACHE II	23 (18, 28)	29 (23, 33)	< 0.001	26 (19, 31)	23 (20, 30)	0.557
SOFA	8 (6, 10)	11 (8, 11)	< 0.001	9 (7, 12)	9 (6, 12)	0.630
mNUTRIC	5 (3, 6)	7 (6, 8)	< 0.001	6 (4, 7)	6 (4, 7)	0.266
mNUTRIC ≥ 6 (high-risk)	68 [40.5]	69 [82.1]	< 0.001	64 [60.4]	73 [50.0]	0.103
Malnutrition	38 [22.6]	28 [33.3]	0.048	31 [29.2]	35 [24.0]	0.347
Admission reasons			< 0.001			0.020
Cardiovascular	11 [6.5]	26 [31.0]		19 [17.9]	18 [12.3]	
Respiratory	31 [18.5]	14 [16.7]		24 [22.6]	21 [14.4]	
Sepsis	48 [28.6]	27 [32.1]		36 [34.0]	39 [26.7]	
Trauma	8 [4.8]	0 [0.0]		4 [3.3]	4 [2.7]	
Metabolic/renal	4 [2.4]	0 [0.0]		2 [1.9]	2 [1.4]	
Gastrointestinal	8 [4.8]	3 [3.6]		3 [2.8]	8 [5.5]	
Post operation	7 [4.2]	0 [0.0]		4 [3.8]	3 [2.1]	
Orthopaedics	3 [1.8]	0 [0.0]		1 [0.9]	2 [1.4]	
Neurological	48 [28.6]	14 [16.7]		13 [12.3]	49 [33.6]	
CPR before ICU adm	11 [6.5]	25 [29.8]	< 0.001	18 [17.0]	18 [12.3]	0.297
Length of MV (days)	4.0 (2.0, 8.0)	5.0 (3.0, 9.0)	0.111	3.0 (2.0, 4.0)	7.0 (4.0, 13.0)	< 0.001
ICU LOS (days)	4.0 (2.0, 8.0)	4.0 (3.0, 8.0)	0.327	3.0 (2.0, 4.0)	7.0 (4.0, 12.0)	< 0.001
Hospital LOS (days)	24.0 (13.5, 43.5)	10.0 (4.0, 16.0)	< 0.001	8.0 (4.0, 15.3)	24.0 (16.0, 45.0)	< 0.001

Values are mean (SD), median (*q*1, *q*3), or count [percentage]. adm, admission; APACHE II, Acute Physiology and Chronic Health Evaluation II; BMI, body mass index; CPR, cardiopulmonary resuscitation; ED, emergency department; ENS, exclusive nutrition support; HD, high dependency; ICU, intensive care unit; LOS, length of stay; MV, mechanical ventilation; mNUTRIC, modified Nutrition Risk in Critically Ill score; OT, operation theatre; SOFA, Sequential Organ Failure Assessment

**Fig. 2:**
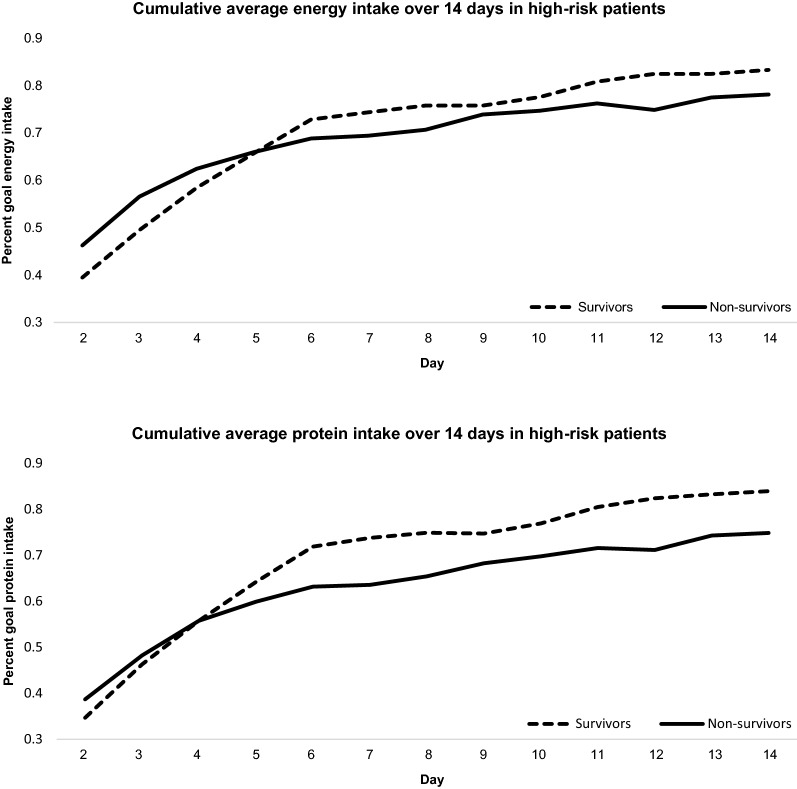
Cumulative average percentage of goal energy and protein intake in 28-day survivors and non-survivors with high risk: defined by the modified Nutrition Risk in Critically Ill (mNUTRIC) score

**Fig. 3:**
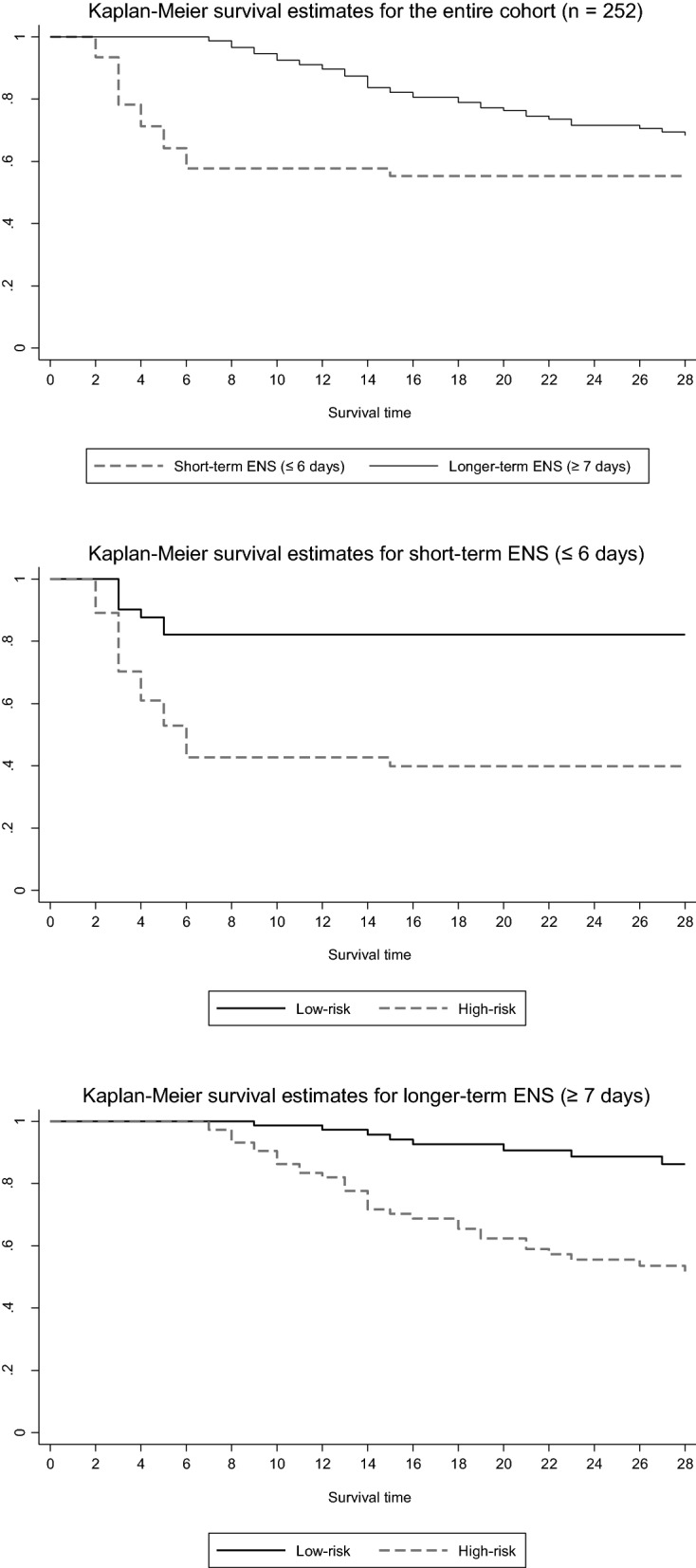
Kaplan–Meier survival estimates stratified by short- and longer-term exclusive nutrition support as well as low- and high-risk patients

The cut-off intervals that defined “short-term ENS” and “longer-term ENS” were set at ≤ 6-days (*n* = 106) and ≥ 7-days (*n* = 146) of ENS, respectively, as clear separation was observed between the per cent goal energy and protein intake of survivors and non-survivors at the univariate level (Fig. 2). In patients with short-term ENS and classified as high-risk (*n* = 64), a large proportion of them perished in the first 6 days of ENS (Fig. 3), and enteral and/or parenteral feeding was ceased due to quick progression to oral feeding or early death. In addition, a higher proportion of them were admitted for medical reasons compared to patients with longer-term ENS. However, the median mNUTRIC score and the proportion of high-risk patients between both groups were not significantly different (Table 1).

The mean (SD) per cent goal energy and protein intake was 65.3% (24.7) [*16.6 (7.0)* kcal/kg] and 61.2% (27.4) [*0.71 (0.34)* g/kg], respectively. To ensure a valid comparison for the associations between energy (and protein) intake and 28-day mortality in patients with short- and longer-term ENS, only the first 6 days of ENS in both groups were assessed. Patients with short-term ENS had significantly lower energy [48.0% vs. 68.7%, *p* value < 0.001 (*12.0* kcal/kg vs. *17.5* kcal/kg] and protein intake [41.6% vs. 64.8% versus, *p* value < 0.001 (*0.47* g/kg vs. *0.77* g/kg], and higher incidence of feeding intolerance when compared to those with longer-term ENS (*p* = 0.001) (Table 2). In addition, patients who required longer-term ENS had a significantly higher percentage of energy provided in the form of protein compared to patients with short-term ENS (17.6% vs. 14.8%, *p* value < 0.001).

**Table 2:** Comparison of the mode of feeding, source, goal, and achieved energy and protein intake between 28-day survivors and non-survivors stratified by days of exclusive nutrition support

Nutrition parameters	Short-term exclusive nutrition support (≤ 6 days)			Longer-term exclusive nutrition support (≥ 7 days)		
	Survivors (*n* = 62)	Non-survivors (*n* = 44)	*p*	Survivors (*n* = 106)	Non-survivors (*n* = 40)	*p*
Mode of feeding						
Enteral	60 [96.8]	42 [95.5]	0.725	92 [86.8]	31 [77.5]	0.169
Parenteral	1 [1.6]	0 [0.0]	0.397	8 [7.5]	4 [10.0]	0.630
Combination	1 [1.6]	2 [4.5]	0.370	6 [5.7]	5 [12.5]	0.163
Energy						
Goal (kcal/kg)	25.5 (5.5)	25.9 (6.3)	0.680	25.9 (4.4)	24.8 (4.3)	0.166
Actual intake (kcal/kg)	10.0 (6.0)	15.0 (6.4)	< 0.001	17.7 (5.2)	16.9 (5.7)	0.389
Actual intake (% goal/kg)	40.0 (22.4)	59.2 (24.1)	< 0.001	68.8 (17.8)	68.2 (19.5)	0.857
Energy sources (%)						
Enteral	82.2 (24.9)	80.8 (28.7)	0.785	82.6 (29.5)	86.0 (23.3)	0.506
IV dextrose	6.7 (17.5)	11.4 (21.3)	0.215	1.4 (2.9)	1.5 (3.3)	0.769
Propofol	10.1 (14.7)	4.1 (6.2)	0.005	5.2 (6.5)	5.3 (9.4)	0.944
Parenteral	1.0 (7.8)	3.7 (17.2)	0.333	10.8 (29.4)	7.1 (21.8)	0.468
Protein						
Goal (g/kg)	1.14 (0.20)	1.15 (0.26)	0.779	1.20 (0.21)	1.15 (0.26)	0.281
Actual intake (g/kg)	0.39 (0.26)	0.57 (0.30)	0.001	0.79 (0.24)	0.71 (0.28)	0.125
Actual intake (% goal/kg)	34.7 (21.6)	51.4 (26.4)	0.001	66.1 (18.9)	61.6 (19.4)	0.206
Protein sources (%)						
Enteral	98.8 (9.6)	95.5 (20.8)	0.338	88.5 (31.2)	92.3 (23.7)	0.486
Parenteral	1.2 (9.6)	4.5 (20.8)	0.338	11.5 (31.2)	7.7 (23.7)	0.486
Per cent protein energy^a^	14.6 (5.1)	15.1 (5.9)	0.600	17.9 (3.5)	16.8 (3.8)	0.100
Fed ≤ 48 h of ICU adm	59 [95.2]	43 [97.7]	0.495	101 [95.3]	37 [92.5]	0.510
Days on ENS	3.0 (3.0, 4.0)	3.0 (3.0, 5.0)	0.735	14.0 (12.0, 14.0)	14.0 (9.3, 14.0)	0.126
Blood glucose (mmol/L)^b^	8.7 (2.9)	8.8 (2.3)	0.846	8.7 (2.3)	9.1 (2.3)	0.345
GRV > 200 mL^c^	0.0 (0.0, 0.3)	0.3 (0.0, 0.7)	0.001	0.2 (0.0, 0.5)	0.0 (0.0, 0.3)	0.140
Hypoglycaemia^c,d^	0.0 (0.0, 0.0)	0.0 (0.0, 0.3)	0.420	0.2 (0.0, 0.5)	0.0 (0.0, 0.3)	0.440

Values are mean (SD), median (q1, q3), or count [percentage]

adm, admission; ENS, exclusive nutrition support; GRV, gastric residual volume; ICU, intensive care unit; IV, intravenous; mNUTRIC, modified Nutrition Risk in Critically Ill score

^a^Percentage of energy provided by protein relative to the total energy intake

^b^Average of daily measurements at 8 am on exclusive nutrition support

^c^Episodes per day on exclusive nutrition support

^d^Blood glucose < 4.0 mmol/L

### Association between energy intake and 28-day mortality during the first 6 days of ENS

Given the crossover associations between per cent goal energy (and protein) intake and 28-day mortality at the univariate level, these associations were adjusted for covariates in multivariable Cox proportional hazard regressions (Table 3). Covariables in the Cox models include days on ENS because energy (and protein) intake increase with time [25, 26] and adjustment for this immortal time bias is recommended and widely practised [9, 11, 25].

**Table 3:** Association between energy (and protein) intake and 28-day mortality in low- and high-risk patients stratified by days on exclusive nutrition support

Energy/protein intake	Short-term exclusive nutrition support (≤ 6 days)			Longer-term exclusive nutrition support (≥ 7 days)		
	Low risk^a^ (*n* = 42)	High risk^a^ (*n* = 64)	Interaction	Low risk^a^ (*n* = 73)	High risk^a^ (*n* = 73)	Interaction
Energy intake (each 10% of goal)	0.93 (0.67, 1.28) *p* = 0.657	1.37 (1.17, 1.61) *p* < 0.001	*p* = 0.280	1.18 (0.75, 1.84) *p* =0.474	0.87 (0.73, 1.04) *p* =0.135	*p* = 0.127
Protein intake (each 10% of goal)	0.97 (0.70, 1.33) *p* =0.846	1.31 (1.10, 1.56) *p* =0.002	*p* = 0.405	1.02 (0.69, 1.51) *p* =0.913	0.78 (0.66, 0.93) *p* =0.006	*p* = 0.088

Values are hazard ratio (95% CI) adjusted for exposure to cardiopulmonary resuscitation before admission to the intensive care unit, nutritional status, and days on exclusive nutrition support

^a^ Low and high risk is defined as scores “0–5” and “6–9” of the modified Nutrition Risk in Critically Ill (mNUTRIC) score, respectively [9]

In the first analysis set (*n* = 252), where timing and dose of nutrition support were not examined, there was no significant association between each 10% increase in goal energy intake and 28-day mortality in high-risk patients [adj-HR 1.22 (95% CI 0.98, 1.53), *p* value: 0.081, interaction between mNUTRIC categories: 0.985].

**Fig. 4:**
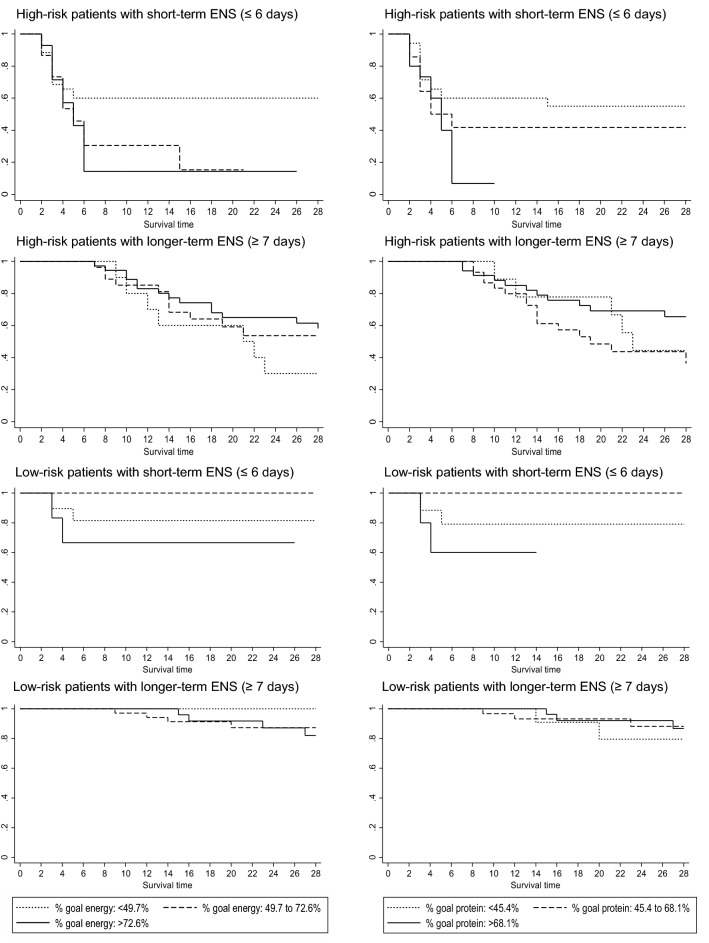
Kaplan–Meier survival estimates of the associations between per cent goal energy (and protein) intake stratified by tertiles and 28-day mortality in low- and high-risk patients with short- and longer-term exclusive nutrition support

In the second analysis set, examining the effects of timing and dose of nutrition support, both univariate and multivariable analyses were performed to determine the associations between per cent goal energy intake and 28-day mortality in low- and high-risk patients with short- and longer-term ENS. *Univariate analyses*—per cent goal energy intake was divided into tertiles, and the associations with 28-day mortality in low- and high-risk patient with short- and longer-term ENS are illustrated in Fig. 4. In low- and high-risk patients with short-term ENS, goal energy intake in the highest tertiles was associated with the highest 28-day mortality risk. In contrast, goal energy intake in the highest tertile was associated with the lowest mortality risk in high-risk patients with longer-term ENS. *Multivariable analyses*—in patients with short-term ENS, there was no significant interaction in the group (*p* value 0.280) (Table 3). However, high-risk patients had a 37% higher hazard (*p* value < 0.001) of 28-day mortality with each 10% increase in goal energy intake, while low-risk patients lost significance. Similarly, there was no significant interaction (*p* value 0.127) in patients with longer-term ENS. While there was an inverse association between per cent goal energy intake and 28-day mortality in high-risk patients, this was not statistically significant (*p* value 0.135).

### Association between energy intake and 28-day mortality in patients with up to 14 days of ENS

There were significant interactions between mNUTRIC categories (*p* value 0.034), but the association between per cent goal energy intake and 28-day mortality in both the mNUTRIC categories was not significant. Every 10% increase in goal energy intake was associated with a nonsignificant increased hazard of 28-day mortality in low-risk patients [adj. HR 1.18 (95% CI 0.83, 4.82), *p* value 0.122], whereas this was inverse in high-risk patients [adj-HR 0.88 (95% CI 0.70,1.09), *p* value 0.234].

### Association between protein intake and 28-day mortality during the first 6 days of ENS

There was no significant association between protein intake and 28-day mortality in high-risk patients when timing and dose of nutrition support were not examined [adj-HR for each 10% increase in goal protein intake for the entire cohort (*n* = 252): 1.14 (95% CI 0.93, 1.39), *p* value: 0.231, interaction between mNUTRIC categories: 0.881].

In the second analysis set, where the effects of timing and dose of nutrition support were examined, both univariate and multivariable analyses were performed to determine whether the associations between per cent goal protein intake and 28-day mortality in patients with low and high risk were stratified by short- and longer-term ENS. *Univariate analyses*—per cent goal protein intake was divided into tertiles, and the associations with 28-day mortality in low- and high-risk patient with short- and longer-term ENS are illustrated in Fig. 4. In low- and high-risk patients with short-term ENS, goal protein intake in the highest tertiles was associated with the highest 28-day mortality risk. In contrast, goal protein intake in the highest tertile was associated with the lowest mortality risk in high-risk patients with longer-term ENS. *Multivariable analyses*—in patients with short-term ENS, there was no significant interaction in the group (Table 3). However, high-risk patients had a 31% higher hazard of 28-day mortality with each 10% increase in goal protein intake (*p* value 0.002). In patients with longer-term ENS, the association between per cent goal protein intake and 28-day mortality varied by mNUTRIC categories with a trend of interactions (*p* value: 0.088): High-risk patients had a 22% lower hazard of 28-day mortality with each 10% increase in goal protein intake (*p* value 0.006).

### Association between protein intake and 28-day mortality in patients with up to 14 days of ENS

The association between per cent goal protein intake and 28-day mortality varied by mNUTRIC categories (interaction *p* value: 0.029), such that high-risk patients had a 19% lower hazard of 28-day mortality with each 10% increase in goal protein intake [adj-HR 0.81 (95% CI 0.67, 0.99), *p* value 0.036]. However, this association was not present in low-risk patients [adj-HR 1.23 (95% CI 0.78, 1.97), *p* value 0.375].

## Discussion

To our knowledge, this is the first study to suggest that the association between mNUTRIC score and 28-day mortality can be modified by the timing and dose of nutrition support. In high-risk patients with short-term ENS (≤ 6 days), energy (and protein) intake was positively associated with 28-day mortality risk. In contrast, protein intake was inversely associated with 28-day mortality in high-risk patients who required longer-term ENS (≥ 7 days).

### Association between energy intake and 28-day mortality

The average energy intake achieved in our study was similar to three previous studies that determined the validity of the mNUTRIC score (58.5–64.5% of energy goal) [11, 13, 27] but we had different observations. When our cohort was analysed in its entirety (irrespective of timing and dose of nutrition support), energy intake was not associated with 28-day mortality. These findings are consistent with previous work by Arabi et al. [13] but differ those from Rahman et al. [9]. The reasons for this lack of concordance are unclear, but the latter was conducted as a post hoc analysis of a multicentre randomised controlled trial (RCT) originally undertaken to examine the effects of glutamine and antioxidant supplementation in critically ill patients [28]. In the post hoc analysis, the investigators examined the external validity of the mNUTRIC score in identifying those patients who will benefit most from adequate energy intake. It demonstrated an inverse association between energy intake and 28-day mortality in high-risk patients. However, the lack of statistical adjustment for the amount of glutamine intake may limit the interpretation of this finding as it is possible that adequate energy, when combined with glutamine, may result in lower mortality per se [29, 30]. This potential confounder was avoided in the post hoc analysis conducted by Arabi et al. [13] since the original study [4] was specifically designed to examine the effects of energy intake on mortality. Hence, Arabi’s findings of the lack of significant association between energy intake and 28-day mortality [OR 0.93 (95% CI 0.60, 1.44), *p* value: 0.74] are more likely to be reliable [13], and our study concurs with this result. It is also possible that analysis of the timing and dose of energy intake in that study may also show an inverse association between energy intake and risk of 28-day mortality in high-risk patients receiving longer-term nutrition support.

When timing and dose of nutrition support were considered in our analysis, a trend was observed towards an inverse association between energy intake and mortality risk in high-risk patients who had up to 14 days of ENS. We hypothesise the lack of significance could reflect the small sample size. This trend of inverse association is in agreement with the findings by Compher et al. [11]. In this large multinational prospective cohort study, energy intake was shown to be inversely associated with 60-day mortality in high-risk patients who had up to 12 days of ENS. Consequently, the investigators recommended that all high-risk patients should receive early aggressive nutrition support as they will benefit most from near-goal energy intake [11]. However, as the present study suggested a positive association between early high energy intake and 28-day mortality in high-risk patients with short-term ENS, this recommendation may need to be applied with caution at the early stage of nutrition support.

Furthermore, some studies suggested that early high energy intake is associated with increased mortality in certain groups of patients. A recent RCT (*n* = 78) demonstrated that high energy intake in the first 7 days of ARDS diagnosis resulted in higher mortality [6], and an energy threshold of 18 kcal/kg was significantly associated with mortality in the post hoc analysis [17]. These findings were supported by a larger cohort study (*n* = 298) that included ARDS patients with higher mNUTRIC characteristics [18]. In addition, Arabi et al. [31] also demonstrated that early (≤ 12 days of ENS) high energy intake in a heterogeneous ICU population was significantly associated with 90-day mortality. Collectively, these studies suggested that high energy intake in the early stage of nutrition support may not be of benefit in all high-risk patients. However, the mechanism of harm associated with early high energy intake is poorly studied. Some would attribute to mitochondrial toxicity caused by an oversupply of glucose and lipid [32], while others have linked it to the suppression of autophagy [33, 34].

### Association between protein intake and 28-day mortality

Although the average protein goal achieved in our study was similar to two previous studies that determined the validity of the mNUTRIC score (56.5% and 58.9% of protein goal) [11, 27], we observed different results. In patients with short-term ENS, early higher protein intake was associated with increased mortality risk. It is, however, unclear whether this risk is solely attributed to protein or a reflection of the harm associated with early higher energy intake. A recent study suggested that early high protein intake (> 0.8 g/kg) was associated with a higher hazard of 6-month mortality when compared to patients who had protein restriction during the first 3 days of ENS and thereafter a higher protein intake [35]. The average proportion of energy provided by protein (protein–energy) in the enteral feeds used in our hospital is 15%. This level of protein–energy coincided with that received in patients with short-term ENS (Table 2), suggesting that protein modular was minimally used. Since it is impossible to statistically separate protein from energy in the analyses, it will be challenging to differentiate the associations between mortality and protein (and energy) intake.

In contrast, the protein–energy intake in patients with longer-term ENS was significantly higher than those with short-term ENS, suggesting that protein modular was used to increase the protein–energy ratio. This suggests that the inverse association between protein intake and mortality risk in patients with longer-term ENS is more likely, a result concordant with earlier studies [11].

### Strength and limitations

There are some advantages in the current study. The consecutive recruitment, complete follow-up, and blinding of the treatment team to the objectives of the study minimised the selection, attrition, and treatment biases. In addition, the exclusion of moribund patients reduced the artificial inflation of the association between 28-day mortality and inadequate nutrition support—since these patients generally receive little nutrition due to poor tolerance or comfort feeding, and death is mainly due to disease severity.

There are, however, several limitations that must be considered before drawing conclusions with clinical implications. This was a single-centre observational study with a small sample of heterogeneous patients. The positive association between early high energy intake and 28-day mortality in high-risk patients was not expected. By indication, severely ill patients (hence short survival time) would usually receive lesser nutrition due to enteral feeding intolerance, and this may artificially inflate the inverse association between nutrition intake and mortality. However, our results were in the opposite direction despite our best efforts to adjust for known confounders. Therefore, the presence of residual confounders inherently limits our results to associations rather than causation. In addition, it was beyond the scope of our study to investigate possible causes for this observation. Therefore, our study should be considered as hypothesis generating and require further confirmation from larger or more comprehensive studies that consider extensive number of confounders.

### Future research and implications for practice

In our study, energy and protein intake was positively associated with 28-day mortality in high-risk patients with short-term ENS, while it was not associated with 28-day mortality in the group with longer-term ENS. These results are disconcerting because the mNUTRIC score did not discriminate these two groups of patients (median mNUTRIC scores for both groups were 6), and it may not be possible for clinicians to accurately predict the length of ENS at ICU admission. Some characteristics of patients with short- and longer-term ENS are given in Table 1. Of note, a higher proportion of patients with short-term ENS were admitted for medical reasons, and more had cardiovascular or respiratory issues as compared to those with longer-term ENS. Conversely, patients who required longer-term ENS were those admitted with neurological issues. Therefore, there is a need to identify subgroups of patients who would likely benefit or be harmed by early aggressive nutrition support.

The present study supports the requirement for larger confirmatory studies to further investigate the modifying effect of timing and dose of nutrition support in high-risk patients. Ideally, this could be achieved by identifying biomarkers that define the different phases (i.e. acute, sub-acute, and chronic) of critical illness [36] and testing whether limiting and increasing energy and protein intake at different phases would be beneficial. Until this is achieved, we suggest a prudent approach to nutrition support. The energy and protein intake that was associated with identical mortality risks in patients with short- and longer-term ENS was 50% of goal (Additional file 1: Figure S1). Therefore, to achieve equipoise, clinicians may feed high-risk patients at 50% of the energy and protein goal in the early periods of ICU admission and intensify the provision of nutrition if ENS was required for a more extended period.

## Conclusion

A modifying effect of timing and dose of nutrition support may be present in some high-risk patients where higher energy intake at the early phase of nutrition support was associated with higher 28-day mortality. Given the lack of parameters that would determine high-risk patients’ response to early high energy intake, the need for future studies cannot be overemphasised.

## Additional file

**Additional file 1.** Associations between goal energy (and protein) intake and 28-day mortality by multivariable logistic regressions.

## Abbreviations

adj-HR: adjusted hazard ratio
adm: admission
APACHE II: Acute Physiology and Chronic Health Evaluation II
BMI: body mass index
CPR: cardiopulmonary resuscitation
ED: emergency department
ENS: exclusive nutrition support
GRV: gastric residual volume
HD: high dependency
ICU: intensive care unit
LOS: length of stay
MV: mechanical ventilation
mNUTRIC: modified Nutrition Risk in Critically Ill
OT: operation theatre
SOFA: Sequential Organ Failure Assessment

## References

[1] Patel JJ, Martindale RG, McClave SA (2017). Controversies surrounding critical care nutrition: an appraisal of permissive underfeeding, protein, and outcomes. JPEN J Parenter Enteral Nutr.

[2] Casaer MP, Mesotten D, Hermans G, Wouters PJ, Schetz M, Meyfroidt G (2011). Early versus late parenteral nutrition in critically ill adults. N Engl J Med.

[3] Casaer MP, Wilmer A, Hermans G, Wouters PJ, Mesotten D, Van den Berghe G (2013). Role of disease and macronutrient dose in the randomized controlled EPaNIC trial: a post hoc analysis. Am J Respir Crit Care Med.

[4] Arabi YM, Aldawood AS, Haddad SH, Al-Dorzi HM, Tamim HM, Jones G (2015). Permissive underfeeding or standard enteral feeding in critically ill adults. N Engl J Med.

[5] Arabi YM, Tamim HM, Dhar GS, Al-Dawood A, Al-Sultan M, Sakkijha MH (2011). Permissive underfeeding and intensive insulin therapy in critically ill patients: a randomized controlled trial. Am J Clin Nutr.

[6] Braunschweig CA, Sheean PM, Peterson SJ, Gomez Perez S, Freels S, Lateef O (2015). Intensive nutrition in acute lung injury: a clinical trial (INTACT). JPEN J Parenter Enteral Nutr.

[7] Doig GS, Simpson F, Sweetman EA, Finfer SR, Cooper DJ, Heighes PT (2013). Early parenteral nutrition in critically ill patients with short-term relative contraindications to early enteral nutrition: a randomized controlled trial. JAMA.

[8] Petros S, Horbach M, Seidel F, Weidhase L (2016). Hypocaloric vs normocaloric nutrition in critically ill patients: a prospective randomized pilot trial. JPEN J Parenter Enteral Nutr.

[9] Rahman A, Hasan RM, Agarwala R, Martin C, Day AG, Heyland DK (2016). Identifying critically-ill patients who will benefit most from nutritional therapy: further validation of the “modified NUTRIC” nutritional risk assessment tool. Clin Nutr.

[10] Heyland DK, Dhaliwal R, Jiang X, Day AG (2011). Identifying critically ill patients who benefit the most from nutrition therapy: the development and initial validation of a novel risk assessment tool. Crit Care.

[11] Compher C, Chittams J, Sammarco T, Nicolo M, Heyland DK (2017). Greater protein and energy intake may be associated with improved mortality in higher risk critically ill patients: a multicenter, multinational observational study. Crit Care Med.

[12] Mukhopadhyay A, Henry J, Ong V, Leong CS-F, Teh AL, van Dam RM (2017). Association of modified NUTRIC score with 28-day mortality in critically ill patients. Clin Nutr.

[13] Arabi YM, Aldawood AS, Al-Dorzi HM, Tamim HM, Haddad SH, Jones G (2017). Permissive underfeeding or standard enteral feeding in high- and low-nutritional-risk critically ill adults. Post hoc analysis of the PermiT Trial. Am J Respir Crit Care Med.

[14] Desachy A, Clavel M, Vuagnat A, Normand S, Gissot V, Francois B (2008). Initial efficacy and tolerability of early enteral nutrition with immediate or gradual introduction in intubated patients. Intensive Care Med.

[15] Heyland DK, Cahill NE, Dhaliwal R, Wang M, Day AG, Alenzi A (2010). Enhanced protein-energy provision via the enteral route in critically ill patients: a single center feasibility trial of the PEP uP protocol. Crit Care.

[16] Heidegger CP, Berger MM, Graf S, Zingg W, Darmon P, Costanza MC (2013). Optimisation of energy provision with supplemental parenteral nutrition in critically ill patients: a randomised controlled clinical trial. Lancet.

[17] Braunschweig CL, Freels S, Sheean PM, Peterson SJ, Perez SG, McKeever L (2017). Role of timing and dose of energy received in patients with acute lung injury on mortality in the Intensive Nutrition in Acute Lung Injury Trial (INTACT): a post hoc analysis. Am J Clin Nutr.

[18] Peterson SJ, Lateef OB, Freels S, McKeever L, Fantuzzi G, Braunschweig CA (2017). Early exposure to recommended calorie delivery in the intensive care unit is associated with increased mortality in patients with acute respiratory distress syndrome. JPEN J Parenter Enteral Nutr.

[19] Doig GS, Simpson F, Heighes PT, Bellomo R, Chesher D, Caterson ID (2015). Restricted versus continued standard caloric intake during the management of refeeding syndrome in critically ill adults: a randomised, parallel-group, multicentre, single-blind controlled trial. Lancet Respir Med.

[20] Detsky AS, McLaughlin JR, Baker JP, Johnston N, Whittaker S, Mendelson RA (1987). What is subjective global assessment of nutritional status?. JPEN J Parenter Enteral Nutr.

[21] Lew CCH, Cheung KP, Chong MFF, Chua AP, Fraser RJL, Miller M (2017). Combining 2 commonly adopted nutrition instruments in the critical care setting is superior to administering either one alone. JPEN J Parenter Enteral Nutr.

[22] Lew CCH, Yandell R, Fraser RJ, Chua AP, Chong MFF, Miller M (2017). Association between malnutrition and clinical outcomes in the intensive care unit: a systematic review. JPEN J Parenter Enteral Nutr.

[23] Lew CCH, Wong GJY, Cheung KP, Chua AP, Chong MFF, Miller M (2017). Association between malnutrition and 28-day mortality and intensive care length-of-stay in the critically ill: a prospective cohort study. Nutrients.

[24] Krenitsky J (2005). Adjusted body weight, pro: evidence to support the use of adjusted body weight in calculating calorie requirements. Nutr Clin Pract.

[25] Heyland DK, Cahill N, Day AG (2011). Optimal amount of calories for critically ill patients: depends on how you slice the cake!. Crit Care Med.

[26] Bellomo R, Cass A, Cole L, Finfer S, Gallagher M, Lee J (2014). Calorie intake and patient outcomes in severe acute kidney injury: findings from The Randomized Evaluation of Normal vs. Augmented Level of Replacement Therapy (RENAL) study trial. Crit Care.

[27] Lee ZY, Noor Airini I, Barakatun-Nisak MY (2018). Relationship of energy and protein adequacy with 60-day mortality in mechanically ventilated critically ill patients: A prospective observational study. Clin Nutr..

[28] Heyland D, Muscedere J, Wischmeyer PE, Cook D, Jones G, Albert M (2013). A randomized trial of glutamine and antioxidants in critically ill patients. N Engl J Med.

[29] Pocock SJ, Assmann SE, Enos LE, Kasten LE (2002). Subgroup analysis, covariate adjustment and baseline comparisons in clinical trial reporting: current practice and problems. Stat Med.

[30] Preiser J-C, Wernerman J (2013). REDOXs important answers, many more questions raised!. JPEN J Parenter Enteral Nutr.

[31] Arabi YM, Tamim HM, Sadat M (2017). Reply to: “Letter to the editor regarding permissive underfeeding or standard enteral feeding in high-and low-nutritional risk critically ill patients”. Am J Respir Crit Care Med.

[32] Picard M, Wallace DC, Burelle Y (2016). The rise of mitochondria in medicine. Mitochondrion.

[33] Marik PE (2016). Is early starvation beneficial for the critically ill patient?. Curr Opin Clin Nutr Metab Care.

[34] Ingels C, Vanhorebeek I, Van den Berghe G (2018). Glucose homeostasis, nutrition and infections during critical illness. Clin Microbiol Infect.

[35] Koekkoek W, van Setten CHC, Olthof LE, Kars J, van Zanten ARH (2018). Timing of PROTein INtake and clinical outcomes of adult critically ill patients on prolonged mechanical VENTilation: the PROTINVENT retrospective study. Clin Nutr.

[36] Arabi YM, Casaer MP, Chapman M, Heyland DK, Ichai C, Marik PE (2017). The intensive care medicine research agenda in nutrition and metabolism. Intensive Care Med.

