# Supplementary material for: When timing and dose of nutrition support were examined, the modified Nutrition Risk in Critically Ill (mNUTRIC) score did not differentiate high-risk patients who would derive the most benefit from nutrition support: a prospective cohort study
Source: Ann Intensive Care. 2018 Oct 22;8:98. doi: 10.1186/s13613-018-0443-1 (PMC6197342; doi:10.1186/s13613-018-0443-1)
Supplement: Supplementary file 1 — Additional file 1. Associations between goal energy (and protein) intake and 28-day mortality by multivariable logistic regressions. [file 13613_2018_443_MOESM1_ESM.docx]

| **Table S1: Association between energy (and protein) intake and 28-day mortality in low- and high-risk patients stratified by days on exclusive nutrition support** | | | | | | | |
| --- | --- | --- | --- | --- | --- | --- | --- |
| **Energy/ Protein** | **Short-term exclusive nutrition support (≤ 6 days)** | | |  | **Longer-term exclusive nutrition support (≥ 7 days)** | | |
| **intake** | **Low-risk ^a^ (n=42)** | **High-risk ^a^ (n=64)** | **Interaction** |  | **Low-risk ^a^ (n=73)** | **High-risk ^a^ (n=73)** | **Interaction** |
|  |  |  |  |  |  |  |  |
| **Energy intake** | 1.01 (0.67, 1.41) | 1.91 (1.31, 2.80) | *p* = 0.069 |  | 1.25 (0.75, 2.07) | 0.85 (0.65, 1.11) | *p* = 0.109 |
| **(each 10% of goal)** | *p* = 0.966 | *p* = 0.001 |  |  | *p =* 0.396 | *p =* 0.227 |  |
|  | HL-GOF = 0.384 | HL-GOF = 0.433 |  |  | HL-GOF = 0.815 | HL-GOF = 0.126 |  |
|  | R^2^ = 0.356 | R^2^ = 0.457 |  |  | R^2^ = 0.128 | R^2^ = 0.174 |  |
|  |  |  |  |  |  |  |  |
| **Protein intake** | 1.04 (0.69, 1.56) | 1.54 (1.15, 2.05) | *p* = 0.209 |  | 1.03 (0.66, 1.59) | 0.68 (0.50, 0.93) | *p* = 0.059 |
| **(each 10% of goal)** | *p =* 0.845 | *p =* 0.004 |  |  | *p =* 0.904 | *p =* 0.015 |  |
|  | HL-GOF = 0.423 | HL-GOF = 0.387 |  |  | HL-GOF = 0.561 | HL-GOF = 0.020 |  |
|  | R^2^ = 0.357 | R^2^ = 0.357 |  |  | R^2^ = 0.108 | R^2^ = 0.258 |  |
|  |  |  |  |  |  |  |  |
| Values are adjusted odds ratio (95% CI) adjusted for exposure to cardiopulmonary resuscitation before admission to the intensive care unit, nutritional status, | | | | | | | |
| and days on exclusive nutrition support | | | | | | | |
| HL-GOF: Hosmer-Lemeshow goodness of fit, R^2^: Max-rescaled R-square | | | | | | | |
| ^a:^ Low- and high-risk are defined as scores “0-5” and “6-9” of the modified Nutrition Risk in Critically ill (mNUTRIC) score respectively [9] | | | | | | | |

*p* = 0.001

*p* = 0.966

A Interactions: *p* = 0.069

*p* = 0.277

*p* = 0.396

B Interactions: *p* = 0.109

*p* = 0.004

*p* = 0.845

A Interactions: *p* = 0.209

*p* = 0.015

*p* = 0.904

B Interactions: *p* = 0.059

Figure S1: Predicted probability of 28-day mortality (adjusted odds ratio with 95% confidence interval as shaded regions) and percent goal energy and protein received during the first 6 days of exclusive nutrition support in patients with low- and high-risk: defined by the modified Nutrition Risk in Critically ill (mNUTRIC) score

**A:** Patients with short-term exclusive nutrition support (≤ 6 days)

**B:** Patients with longer-term exclusive nutrition support (≥ 7 days)
